# Integrated Mindfulness and Cardiopulmonary Rehabilitation Improves Cardiopulmonary Performance and Psychological Stress in Pulmonary Arterial Hypertension

**DOI:** 10.1155/carj/3058088

**Published:** 2026-06-29

**Authors:** Jingjuan Qian, Chunjie Song, Meng Yuan, Yanfeng Bai, Dan Li, Yani Chen

**Affiliations:** ^1^ Suqian First Hospital, Suqian, 223800, China

**Keywords:** cardiopulmonary rehabilitation, mindfulness-based stress reduction, oxygen delivery, psychological stress, pulmonary arterial hypertension

## Abstract

**Objective:**

To investigate the effects of mindfulness‐based stress reduction (MBSR) combined with cardiopulmonary rehabilitation (CR) training on cardiopulmonary function, oxygen delivery efficiency, and psychological stress in patients with pulmonary arterial hypertension (PAH).

**Methods:**

A total of 50 clinically stable PAH patients were randomly assigned to an intervention group (*N* = 25) and a control group (*N* = 25). The intervention group received a 6‐week program combining MBSR and standardized CR, while the control group received conventional health education and standard pharmacological management. Cardiopulmonary exercise capacity, hemodynamic parameters, and psychological stress levels were assessed before and after the intervention.

**Results:**

After 6 weeks, the intervention group showed significantly improved percentage of predicted peak oxygen uptake (84.3 ± 12.1% vs. 71.2 ± 13.4%, *p* = 0.001), oxygen delivery (1650 ± 410 mL/min vs. 1388 ± 375 mL/min, *p* = 0.022), and arterial oxygen content (18.5 ± 2.1 mL/dL vs. 17.1 ± 1.8 mL/dL, *p* = 0.015). Hemodynamically, pulmonary vascular resistance significantly decreased (415 ± 265 vs. 586 ± 310 dyn·s/cm^5^, *p* = 0.040), and pulmonary artery compliance increased (2.12 ± 0.46 mL/mmHg vs. 1.76 ± 0.42 mL/mmHg, *p* = 0.009). In terms of psychological stress, the intervention group showed significantly lower scores in HAMA (11.28 ± 3.94 vs. 14.22 ± 4.06, *p* = 0.013), HAMD (11.36 ± 4.32 vs. 15.26 ± 4.89, *p* = 0.011), and SPBS (18.04 ± 4.83 vs. 23.02 ± 5.44, *p* = 0.001) compared with the control group.

**Conclusion:**

MBSR combined with CR significantly improves cardiopulmonary exercise performance, oxygen transport capacity, and hemodynamic function in PAH patients, while also reducing psychological stress levels. This combined intervention demonstrates good safety and promising clinical applicability.

## 1. Introduction

Pulmonary arterial hypertension (PAH) is a progressive disease characterized by elevated pulmonary arterial pressure, frequently resulting in reduced exercise tolerance, dyspnea, and right heart failure [1, 2] and commonly accompanied by psychological disorders such as anxiety and depression [3, 4]. While traditional treatment strategies have primarily focused on pharmacological therapy, recent years have seen increasing emphasis on nonpharmacological interventions—particularly cardiopulmonary rehabilitation (CR)—for their potential to improve functional status and health‐related quality of life (HRQoL) in this population [5–7].

CR has been shown to significantly enhance cardiopulmonary function in patients with PAH. Multiple studies have reported improvements in exercise capacity and cardiopulmonary performance following structured rehabilitation programs [6, 8–10]. For instance, a prospective randomized trial in PAH patients demonstrated that CR led to improvements in hemodynamic parameters and cardiopulmonary function [11]. Another study suggested that even once‐weekly CR sessions could yield beneficial physical and psychological outcomes in patients with regional cardiopulmonary disease [12]. Furthermore, the impact of CR in patients with chronic thromboembolic pulmonary hypertension (CTEPH) has been evaluated using cardiopulmonary exercise testing (CPET), underscoring its value as both a therapeutic and assessment tool [13]. CPET, in particular, plays a critical role in assessing exercise‐induced cardiopulmonary responses and is instrumental in diagnosing PAH, including exercise‐induced variants [14, 15].

Beyond its physiological benefits, CR has also been shown to yield psychological advantages in patients with PAH [3, 16]. Individuals with chronic diseases, including PAH, often experience comorbid psychological conditions such as anxiety and depression [3, 17]. CR programs typically incorporate exercise training, disease education, and psychological support, constituting a multimodal approach that alleviates psychological burden, enhances emotional regulation, and improves HRQoL [16, 18, 19]. For example, CR has been shown to improve both cardiopulmonary function and arterial blood gas parameters in patients with chronic obstructive pulmonary disease (COPD) complicated by cor pulmonale [20], indicating that its benefits may extend to cardiac function across various forms of pulmonary disease.

Mindfulness‐based interventions, as a form of psychological support, have garnered attention in clinical contexts in recent years. Mindfulness training, including mindfulness meditation, has been shown to improve attention and self‐awareness [21]. Although direct research on the application of mindfulness in PAH is limited, studies have explored its efficacy in reducing psychological stress among healthcare professionals involved in cardiopulmonary resuscitation (CPR), suggesting potential applicability in high‐stress medical environments [21]. Given that psychological stress can adversely affect CPR performance [22], mindfulness may also hold value in stress modulation.

Integrating mindfulness with CR may offer a comprehensive therapeutic framework for PAH management. While CR targets physiological performance and exercise tolerance, mindfulness focuses on emotional regulation and stress reduction. This combined intervention model has the potential to simultaneously improve cardiopulmonary metrics and equip patients with better psychological coping strategies. Empirical evidence suggests that experiential avoidance is correlated with both psychological well‐being and cardiopulmonary endurance [23], underscoring the relevance of psychological variables in rehabilitation outcomes. Through mindfulness practice, patients may develop enhanced acceptance and adaptive responses to disease‐related distress, thereby fostering greater adherence to rehabilitation protocols and maximizing therapeutic gains.

Therefore, this study hypothesized that the integration of mindfulness‐based stress reduction (MBSR) with CR would result in superior improvements in cardiopulmonary performance, oxygen transport efficiency, and psychological well‐being compared with CR alone in patients with PAH. By exploring the synergistic effects of these interventions, this study was designed to provide valuable insights into the clinical management of PAH and offer evidence‐based recommendations for integrating psychological and physical rehabilitation strategies into routine clinical care. This research will contribute to a better understanding of how multidisciplinary approaches can optimize outcomes for PAH patients, enhancing both their physical and emotional health.

## 2. Methods

### 2.1. Study Design and Participants

This was a prospective, randomized controlled clinical trial conducted between June 2023 and June 2024 at Suqian First Hospital. This study was conducted in accordance with the Declaration of Helsinki and relevant national regulations. The protocol was reviewed and approved by the Institutional Review Board/Ethics Committee of Suqian First Hospital (approval no.: IRB‐KY202204‐2022, date: 12th April 2022). All participants were adults and provided written informed consent prior to enrollment and any study procedures. An independent safety monitoring committee adjudicated adverse events; no serious adverse events occurred during the intervention period.

A total of 50 patients diagnosed with PAH were enrolled. The inclusion criteria were as follows: (1) diagnosis of PAH according to established criteria [24]; (2) New York Heart Association (NYHA) functional class II or III; (3) disease duration ≥ 6 months with clinically stable status and no acute exacerbation; (4) age between 18 and 80 years, with the ability to communicate and comply with study procedures; and (5) provision of informed consent.

Exclusion criteria included (1) comorbid severe psychiatric disorders, cognitive impairment, or inability to participate in the intervention; (2) history of acute myocardial infarction, severe hepatic/renal dysfunction, or malignancy; (3) recent treatment with anxiolytics or antidepressants; (4) contraindications to exercise or inability to perform physical activity; and (5) dropout during the intervention or missing data.

The sample size was calculated by using PASS 15.0. Assuming an effect size (Cohen’s d) of 0.9 for improvement in VO2 peak, a two‐sided alpha of 0.05, and a statistical power of 0.8, the minimum required sample size was calculated as 21 participants per group. Allowing for a 15% dropout rate, a total of 50 participants (25 per group) were recruited.

Participants were randomly assigned in a 1:1 ratio to either the intervention or control group using a computer‐generated random number sequence. Allocation concealment was maintained by employing sequentially numbered, opaque, sealed envelopes prepared by an independent statistician not involved in the study implementation. In the intervention group, there were 20 females (80.0%) and 5 males, with a median age of 45 years (range: 21–70) and a median disease duration of 5.0 years (range: 0.8–15.0). In the control group, 19 participants (76.0%) were female, with a median age of 44 years (range: 22–68) and a median disease duration of 4.9 years (range: 0.6–14.5). The distribution of age groups, BMI (28.1 ± 4.3 vs. 27.7 ± 4.8 kg/m^2^), PAH classification (idiopathic, heritable, or CTD associated), standard‐of‐care PAH therapies (prostacyclin infusion and double or triple therapy), and diuretic usage were comparable between groups. No statistically significant differences were observed in any of the baseline characteristics between the intervention and control groups (all *p* > 0.05), indicating that the two groups were well‐matched and comparable at baseline (Table 1). Outcome assessors conducting CPET, right heart catheterization (RHC), and psychological evaluations were blinded to group allocation throughout the study.

**TABLE 1:** Comparison of baseline characteristics and demographic data between the two groups of patients with pulmonary arterial hypertension.

Characteristics	Intervention group (*N* = 25)	Control group (*N* = 25)	Statistics	*p*
Female, *N* (%)	20 (80.0)	19 (76.0)	0.114	0.736
Age, y; median (range)	45 (21–70)	44 (22–68)	−0.356	0.722
Age group, y; *N* (%)			0.309	0.857
18–44	13 (52.0)	12 (48.0)		
45–64	7 (28.0)	8 (32.0)		
65–74	5 (20.0)	5 (20.0)		
≥ 75	0 (0.0)	0 (0.0)		
BMI (kg/m^2^), mean ± SD	28.1 ± 4.3	27.7 ± 4.8	0.280	0.781
Time since diagnosis, y; median (range)	5.0 (0.8–15.0)	4.9 (0.6–14.5)	−0.298	0.766
PAH classification, *N* (%)			0.486	0.784
Idiopathic	17 (68.0)	16 (64.0)		
Heritable	1 (4.0)	1 (4.0)		
Associated with CTD	7 (28.0)	8 (32.0)		
Standard‐of‐care PAH therapy, *N* (%)			0.471	0.791
Prostacyclin infusion therapy	14 (56.0)	13 (52.0)		
Double therapy	12 (48.0)	11 (44.0)		
Triple therapy	13 (52.0)	14 (56.0)		
Use of diuretics, *n* (%)^∗^	17 (68.0)	18 (72.0)	0.098	0.754
Bumetanide	1 (4.0)	1 (4.0)		
Chlorothiazide	2 (8.0)	3 (12.0)		
Furosemide	10 (40.0)	11 (44.0)		
Metolazone	3 (12.0)	2 (8.0)		
Spironolactone	6 (24.0)	5 (20.0)		
Torasemide	4 (16.0)	4 (16.0)		

*Note:* Statistical analyses included the chi‐square test, *t*‐test, and Mann–Whitney *U* test (rank‐sum test). All *p* values were > 0.05, indicating good baseline comparability between the two groups prior to intervention.

Abbreviations: BMI, Body Mass Index; CTD, connective tissue disease; PAH, pulmonary arterial hypertension.

^∗^Diuretic use may involve multiple counting.

### 2.2. Intervention Protocol

The intervention lasted for 6 weeks in both groups. The control group received routine CR training. The intervention group received an integrated program combining MBSR with standard CR.

#### 2.2.1. CR Training

CR sessions were conducted three times per week for six consecutive weeks. Each session lasted approximately 60 min, including a 10‐min warmup, 40 min of structured aerobic and resistance training, and a 10‐min cool‐down period. Training intensity was individualized according to baseline CPET results, targeting 60%–70% of the patient’s peak VO_2_ (or 60%–80% of heart rate reserve) during aerobic segments, corresponding to a perceived exertion of 11–13 on the Borg scale. Continuous monitoring of oxygen saturation (SpO_2_ ≥ 90%), heart rate, and symptoms (Borg dyspnea scale < 5) ensured safety and intensity control.

Each CR session comprised treadmill walking, stationary cycling, and light resistance exercises focusing on major muscle groups. Exercise intensity and workload were progressively increased by 5%–10% weekly based on individual tolerance and physiological responses. Intermittent training was introduced for patients exhibiting exertional dyspnea, allowing alternating 2‐min active and passive recovery phases.

All sessions were conducted under the direct supervision of CR physicians and physiotherapists. Real‐time electrocardiographic and pulse oximetry monitoring were applied throughout training. Any deviations from target parameters triggered immediate adjustment or session termination.

#### 2.2.2. MBSR

Patients in the intervention group participated in MBSR sessions three times per week for 40 min each session, delivered in a group format. The curriculum consisted of the following weekly modules:

 Week 1: Introduction to MBSR and body scan to promote somatic awareness and self‐acceptance.
 Week 2: Mindful breathing with diaphragmatic techniques to improve emotional regulation.
 Week 3: Mindful eating and introspection using sensory‐focused raisin exercises to enhance present‐moment awareness.
 Week 4: Sitting and walking meditation to cultivate attention to surroundings and bodily movements.
 Week 5: Mindful yoga with guided postures to strengthen somatic awareness and emotional regulation.
 Week 6: Consolidation and integration of mindfulness into daily life to support emotional self‐regulation and long‐term psychological adaptation.

MBSR sessions were conducted by certified instructors to ensure standardization and fidelity of delivery. Adherence was tracked via attendance logs and home‐practice diaries. Adequate adherence was defined as ≥ 80% session attendance and ≥ 70% home practice compliance.

### 2.3. CPET

CPET was conducted at baseline and postintervention using a cycle ergometer with an individualized ramp protocol. Measurements included peak oxygen uptake (VO_2_ peak), anaerobic threshold (AT), oxygen pulse, and maximum voluntary ventilation (MVV). Tests were terminated upon achieving respiratory exchange ratio (RER) ≥ 1.10, or upon patient exhaustion. All tests were supervised and interpreted by blinded assessors.

### 2.4. Oxygen Transport Assessment

In all patients, arterial blood samples were obtained at rest and peak exercise. Arterial oxygen content (CaO_2_) was calculated as follows: CaO_2_ = (Hb × 1.34 × SaO_2_) + (0.003 × PaO_2_).

Oxygen delivery (DO_2_) was calculated as follows: DO_2_ = CaO_2_ × cardiac output × 10.

Peak exercise cardiac output was measured directly using RHC with the thermodilution technique during incremental supine cycle ergometry. Three consecutive thermodilution measurements were performed at rest and immediately at peak workload, and the mean of these readings was used for analysis. This approach ensured high accuracy and reproducibility of cardiac output values under exercise conditions.

### 2.5. RHC

All patients underwent RHC at baseline and after 6 weeks. Hemodynamic parameters included mean pulmonary arterial pressure (mPAP), systolic pulmonary arterial pressure (sPAP), mean right atrial pressure (mRAP), pulmonary capillary wedge pressure (PAWP), and pulmonary vascular resistance (PVR), calculated as follows: PVR = (mPAP−PAWP) × 80/cardiac output. Stroke volume derived from RHC (SV_RHC_) was calculated as follows: SV_RHC_ = cardiac output × 1000/heart rate, where cardiac output is expressed in L/min and heart rate in beats/min. All hemodynamic measurements were independently verified by two experienced cardiologists blinded to group allocation. Intraobserver and interobserver coefficients of variation were maintained below 5% across repeated measurements.

### 2.6. Cardiac Magnetic Resonance (CMR) Imaging

CMR was performed using a 1.5 T scanner at baseline and Week 6. Cine sequences in standard short‐axis and long‐axis views were acquired to quantify right ventricle (RV) and left ventricle (LV) volumes and function. Parameters included right ventricular end‐diastolic volume (RVEDV), right ventricular end‐systolic volume (RVESV), RV stroke volume (RVSV), right ventricular ejection fraction (RVEF), RV mass, and analogous LV parameters. Image analysis was performed using CMR42 software by two blinded radiologists.

### 2.7. Psychological Stress Evaluation

Anxiety and depression were assessed at baseline and postintervention using the Hamilton Anxiety Rating Scale (HAMA) [25] and the Hamilton Depression Rating Scale (HAMD) [26]. Psychological burden was measured using the Self‐Perceived Burden Scale (SPBS) [27]. These scales were administered by trained personnel blinded to group allocation.

### 2.8. Safety Monitoring

Adverse events were actively monitored throughout the study. Major events included acute right heart failure, arrhythmia, or hemodynamic collapse, while minor events (e.g., fatigue and musculoskeletal discomfort) were recorded. Events were adjudicated by an independent monitoring committee. No serious adverse events were reported, and both groups showed high adherence and good tolerability.

### 2.9. Statistical Analysis

All randomized participants completed the postintervention assessments. Therefore, analyses were performed on a complete‐case dataset and were operationally equivalent to an intention‐to‐treat approach. Data were analyzed using SPSS Version 24.0 (IBM Corp., Armonk, NY, USA). All randomized participants completed the postintervention assessments. Therefore, analyses were performed on a complete‐case dataset and were operationally equivalent to an intention‐to‐treat approach. Categorical variables were compared using the chi‐square test. Given the pilot sample size, formal effect size estimation was not performed to avoid overinterpretation of imprecise estimates. Continuous variables were tested for normality prior to selecting parametric or nonparametric statistical tests. The magnitude of change and between‐group differences in continuous variables were instead reported as mean ± SD or M50 (M25 and M75) and compared using independent samples *t*‐tests or Mann–Whitney *U* test, which provides a robust descriptive indication of effect direction and clinical relevance. All statistical tests were two‐sided, with a significance level set at *p* < 0.05. Given the pilot design and the absence of significant baseline differences between groups, change scores (post–pre differences) were analyzed without ANCOVA adjustment, which is a statistically valid approach in feasibility trials where randomization ensures baseline equivalence. Besides, within‐group differences were visually represented and summarized descriptively, whereas inferential testing focused on between‐group comparisons of change scores to prevent multiplicity‐related bias.

## 3. Results

### 3.1. Basic Characteristics

A total of 50 patients diagnosed with PAH were enrolled and randomized equally into the intervention group (*N* = 25) and the control group (*N* = 25). Baseline demographic and clinical characteristics are summarized in Table 1. No statistically significant differences were observed between the two groups across all assessed variables (*p* > 0.05), indicating good comparability.

The majority of participants in both groups were female, accounting for 80.0% in the intervention group and 76.0% in the control group (*p* = 0.736). The median age was 45 years (range: 21–70) in the intervention group and 44 years (range: 22–68) in the control group (*p* = 0.722). The distribution of age groups was similar between the groups, with approximately half of the patients under the age of 45.

The mean body mass index (BMI) was comparable between the intervention and control groups (28.1 ± 4.3 kg/m^2^ vs. 27.7 ± 4.8 kg/m^2^, *p* = 0.781). The median time since diagnosis was 5.0 years (range: 0.8–15.0) in the intervention group and 4.9 years (range: 0.6–14.5) in the control group (*p* = 0.766). According to PAH classification, idiopathic PAH was the most common subtype in both groups (68.0% vs. 64.0%), followed by PAH associated with connective tissue disease (28.0% vs. 32.0%), and heritable PAH (4.0% in both groups).

Regarding standard‐of‐care therapies, 56.0% of the patients in the intervention group and 52.0% in the control group were receiving prostacyclin infusion therapy, while 52.0% and 56.0%, respectively, were on triple combination therapy. The use of diuretics was common across both groups (68.0% vs. 72.0%, *p* = 0.754), with furosemide being the most frequently used agent (40.0% vs. 44.0%). Other diuretics included spironolactone, torsemide, and metolazone.

Besides, no statistically significant differences were observed between the intervention group and the control group at baseline, across all assessed hemodynamic, CMR, functional, and laboratory parameters, indicating comparability between groups (all *p* > 0.05, Table 2).

**TABLE 2:** Comparison of cardiac output, stroke volume, and gas exchange–related parameters between the two groups of PAH patients after 6 weeks of intervention (*x* ± *s*).

Characteristics	Intervention group (*N* = 25)	Control group (*N* = 25)	Statistics	*p*
Cardiac output — Resting supine (L/min)	4.83 ± 1.30	4.76 ± 1.22	0.187	0.853
Cardiac output — Resting upright (L/min)	5.12 ± 1.22	5.39 ± 1.56	0.649	0.520
Cardiac output — Peak exercise (L/min)	8.92 ± 2.41	8.74 ± 2.90	0.243	0.809
CO difference (Peak − Upright) (L/min)	3.80 ± 1.71	2.70 ± 1.81	2.327	0.025^∗^
Predicted peak cardiac output (%)	70.6 ± 21.3	66.1 ± 20.9	0.711	0.481
SV_RHC_ — Resting supine (mL/beat)	61.2 ± 18.0	59.0 ± 16.3	0.423	0.674
SV_RHC_ — Resting upright (mL/beat)	62.1 ± 15.1	64.3 ± 19.2	0.446	0.659
SV_RHC_ — Peak exercise (mL/beat)	68.9 ± 19.7	66.5 ± 20.1	0.446	0.659
SV_RHC_ difference (Peak − Upright) (mL/beat)	6.8 ± 14.9	1.3 ± 15.4	2.302	0.027^∗^
Arterial oxygen content (mL/dL)	18.5 ± 2.1	17.1 ± 1.8	2.516	0.015^∗^
Oxygen delivery (mL/min)	1650 ± 410	1388 ± 375	2.378	0.022^∗^
VO_2_ peak as % predicted (%)	84.3 ± 12.1	71.2 ± 13.4	3.505	0.001^∗^
PVR (dyn·s/cm^5^)	415 ± 265	586 ± 310	2.109	0.040^∗^
Pulmonary artery compliance (mL/mmHg)	2.12 ± 0.46	1.76 ± 0.42	2.761	0.009^∗^

^∗^ *p* < 0.05.

Specifically, mPAP was 42.6 ± 14.8 mmHg in the intervention group and 43.4 ± 15.5 mmHg in the control group (*p* = 0.852), while PVR was comparable between groups (642 ± 538 vs. 648 ± 545 dyn·s/cm^5^, *p* = 0.971, Table 3). Measures of right atrial pressure (mRAP), PAWP, and cardiac index were also well balanced (Table 3).

**TABLE 3:** Baseline comparison of hemodynamic parameters, cardiac magnetic resonance imaging, and related indicators in PAH patients (*x* ± *s*).

Characteristics	Intervention group (*N* = 25)	Control group (*N* = 25)	Statistics	*p*
*Hemodynamic parameters*				
PVR (dyn·s/cm^5^)	642 ± 538	648 ± 545	0.037	0.971
mPAP (mmHg)	42.6 ± 14.8	43.4 ± 15.5	0.188	0.852
sPAP (mmHg)	62.9 ± 21.5	63.7 ± 24.1	0.128	0.899
mRAP (mmHg)	3.8 ± 5.9	3.6 ± 6.2	0.114	0.910
PAWP (mmHg)	6.6 ± 3.1	6.4 ± 2.9	0.229	0.820
Cardiac index (L/min/m^2^)	2.82 ± 0.66	2.79 ± 0.72	0.159	0.874

*RV CMR parameters*				
RVEDV (mL)	202.1 ± 53.6	206.4 ± 55.0	0.274	0.785
RVESV (mL)	130.2 ± 52.1	129.7 ± 50.8	0.033	0.974
RVEF (%)	38.6 ± 12.3	37.8 ± 12.7	0.220	0.827
RVSV (mL)	73.2 ± 24.9	75.4 ± 26.1	0.292	0.771
RVSVI (mL/m^2^)	40.2 ± 13.0	41.6 ± 13.6	0.373	0.711
RV mass (g)	208.6 ± 13.8	209.7 ± 15.0	0.267	0.79
RV mass/RVEDV (g/mL)	0.252 ± 0.087	0.254 ± 0.081	0.092	0.927

*LV CMR parameters*				
LVSV (mL)	77.1 ± 22.5	76.6 ± 23.8	0.080	0.936
LVEF (%)	57.2 ± 9.8	57.9 ± 9.5	0.273	0.786
LV mass (g)	94.3 ± 25.7	93.5 ± 26.8	0.113	0.911
RV mass/LV mass	0.56 ± 0.29	0.58 ± 0.28	0.247	0.806

*Others*				
NT‐proBNP (pg/mL)	728 ± 866	740 ± 874	0.053	0.958
6MWD (m)	392 ± 74	388 ± 78	0.190	0.850
Pulse rate (Rest, bpm)	87.8 ± 9.6	88.2 ± 10.2	0.150	0.881
Hb (g/dL)	13.4 ± 1.6	13.2 ± 1.8	0.434	0.666

*Note:* Hb, hemoglobin. All variables were tested for normality. Independent samples *t*‐tests were used for normally distributed data; for nonnormally distributed data, the Mann–Whitney *U* test was applied. All *p* values in the table are > 0.05, indicating no statistically significant differences between groups at baseline and confirming good comparability.

Abbreviations: 6MWD, six‐minute walk distance; EF, ejection fraction; LV, left ventricle; mPAP, mean pulmonary arterial pressure; mRAP, mean right atrial pressure; NT‐proBNP, N‐terminal pro–brain natriuretic peptide; PAWP, pulmonary arterial wedge pressure; PVR, pulmonary vascular resistance; RV, right ventricle; sPAP, systolic pulmonary arterial pressure; SV, stroke volume.

Cardiac MRI‐derived metrics, including RVEDV, RVESV, and RVEF, showed no significant differences. For example, RVEDV was 202.1 ± 53.6 mL in the intervention group and 206.4 ± 55.0 mL in the control group (*p* = 0.785), while RVEF was 38.6% ± 12.3% and 37.8% ± 12.7%, respectively (*p* = 0.827). Similarly, left ventricular parameters such as LV stroke volume (LVSV) and mass were comparable as was the ratio of RV mass to LV mass (0.56 ± 0.29 vs. 0.58 ± 0.28, *p* = 0.806, as shown in Table 3).

Functional capacity at baseline, assessed by the 6‐min walk distance (6MWD), was 392 ± 74 m in the intervention group and 388 ± 78 m in the control group (*p* = 0.850). NT‐proBNP levels were also similar between groups (728 ± 866 vs. 740 ± 874 pg/mL, *p* = 0.958, Table 3) as were resting heart rate and hemoglobin levels.

At baseline, no statistically significant differences were observed between the intervention and control groups in any of the cardiac output or calculated stroke volume (SV_RHC_) parameters, both at rest and during exercise (all *p* > 0.05, Table 4).

**TABLE 4:** Comparison of baseline cardiac output and stroke volume by right heart catheterization in two groups of PAH patients (*x* ± *s*).

Characteristics	Intervention group (*N* = 25)	Control group (*N* = 25)	Statistics	*p*
*Cardiac output (L/min)*				
Resting supine	4.68 ± 1.30	4.72 ± 1.24	0.115	0.909
Resting upright	5.59 ± 1.70	5.52 ± 1.78	0.148	0.883
Peak exercise	8.54 ± 2.92	8.66 ± 3.06	0.146	0.885
Difference between peak exercise and resting upright	3.04 ± 1.88	3.10 ± 1.95	0.113	0.911
Predicted peak cardiac output	64.1 ± 20.7	65.3 ± 20.3	0.207	0.837

*Calculated stroke volume (* *S* *V*_*R* *H* *C*_ *) (mL/beat)*				
Resting supine	57.8 ± 16.1	58.2 ± 15.7	0.089	0.930
Resting upright	68.5 ± 23.7	67.7 ± 22.8	0.134	0.894
Peak exercise	67.1 ± 24.8	66.8 ± 23.9	0.046	0.963
Difference between peak exercise and resting upright	−1.08 ± 14.6	−0.98 ± 15.4	0.025	0.980

*Note:* All variables are presented as mean ± standard deviation (*x* ± *s*). Statistical analysis was performed using the independent samples *t*‐test; for variables not following a normal distribution, the nonparametric Mann–Whitney *U* test was applied.

Specifically, resting supine cardiac output was 4.68 ± 1.30 L/min in the intervention group and 4.72 ± 1.24 L/min in the control group (*p* = 0.909), while upright resting cardiac output was 5.59 ± 1.70 L/min and 5.52 ± 1.78 L/min, respectively (*p* = 0.883). Peak exercise cardiac output was comparable between groups (8.54 ± 2.92 vs. 8.66 ± 3.06 L/min, *p* = 0.885) as was the difference between upright rest and peak exercise (3.04 ± 1.88 vs. 3.10 ± 1.95 L/min, *p* = 0.911). The percentage of predicted peak cardiac output was similarly distributed (64.1% ± 20.7% vs. 65.3% ± 20.3%, *p* = 0.837).

With regard to RHC‐derived stroke volume, the intervention and control groups showed nearly identical resting supine values (57.8 ± 16.1 vs. 58.2 ± 15.7 mL/beat, *p* = 0.930) and upright values (68.5 ± 23.7 vs. 67.7 ± 22.8 mL/beat, *p* = 0.894). Peak exercise SV_RHC_ was also similar (67.1 ± 24.8 vs. 66.8 ± 23.9 mL/beat, *p* = 0.963), with no significant difference in the stroke volume increment from rest to peak between groups (−1.08 ± 14.6 vs. −0.98 ± 15.4 mL/beat, *p* = 0.980).

Together, these findings demonstrate that the two groups were well matched at baseline in terms of global cardiac function and hemodynamic responses to physical activity, providing a robust foundation for evaluating postintervention effects.

### 3.2. Comparison of Major Physiological Indicators Between the Two Groups After 6 Weeks of Intervention

At Week 6 postintervention, both groups exhibited improvements across multiple physiological parameters; however, the intervention group showed significantly greater improvements in key hemodynamic and functional outcomes compared to the control group (Table 5).

**TABLE 5:** Comparison of major physiological indicators between the two PAH groups after 6 weeks of intervention (*x* ± *s*).

Characteristics	Intervention group (*N* = 25)	Control group (*N* = 25)	Statistics	*p*
PVR (dyn·s/cm^5^)	435 ± 270	589 ± 314	2.104	0.040
mPAP (mmHg)	29.2 ± 10.3	37.6 ± 13.4	2.412	0.021
sPAP (mmHg)	43.5 ± 12.3	55.8 ± 17.9	2.363	0.023
mRAP (mmHg)	0.9 ± 1.3	2.1 ± 2.3	3.981	< 0.001
PAWP (mmHg)	3.0 ± 2.1	4.1 ± 2.7	2.093	0.042
Cardiac index (L/min/m^2^)	2.6 ± 0.8	2.2 ± 0.6	2.011	0.049
RVSV (mL)	49.8 ± 20.9	39.3 ± 15.4	2.017	0.048
RVESV (mL)	85.1 ± 39.2	108.2 ± 47.5	2.134	0.038
RVEDV (mL)	134.2 ± 47.6	150.1 ± 52.4	1.291	0.203
RVEF (%)	36.8 ± 12.5	31.2 ± 10.6	1.636	0.109
RVSVI (mL/m^2^)	28.1 ± 9.2	22.6 ± 8.0	2.236	0.032
RV mass (g)	22.8 ± 6.1	24.1 ± 6.7	0.746	0.460
RV mass/RVEDV (g/mL)	0.318 ± 0.082	0.289 ± 0.081	1.323	0.192
LVSV (mL)	66.5 ± 18.9	62.1 ± 21.0	0.747	0.459
LVEF (%)	57.0 ± 9.2	56.3 ± 9.7	0.252	0.802
LV mass (g)	90.2 ± 19.6	92.4 ± 22.5	0.357	0.723
RV mass/LV mass	0.51 ± 0.21	0.54 ± 0.23	0.524	0.604
NT‐proBNP (pg/mL)	198 ± 166	406 ± 212	2.99	0.004
6MWD (m)	458 ± 71	410 ± 66	2.793	0.007
Pulse rate (beats/min)	90.2 ± 10.8	88.5 ± 9.5	0.522	0.604
Hemoglobin (g/dL)	14.8 ± 1.8	14.1 ± 1.5	1.52	0.137

Abbreviations: 6MWD, six‐minute walk distance; mPAP, mean pulmonary arterial pressure; mRAP, mean right atrial pressure; NT‐proBNP, N‐terminal pro–brain natriuretic peptide; PAWP, pulmonary artery wedge pressure; PVR, pulmonary vascular resistance; RVEDV, right ventricular end‐diastolic volume; RVEF, right ventricular ejection fraction; RVSV, right ventricular stroke volume; RVSVI, right ventricular stroke volume index.

In terms of pulmonary hemodynamics, the intervention group demonstrated a significantly lower PVR (435 ± 270 dyn·s/cm^5^) compared with the control group (589 ± 314 dyn·s/cm^5^, *p = *0.040). Both mPAP and sPAP were significantly reduced in the intervention group (*p* = 0.021and *p* = 0.023, respectively). Furthermore, mRAP and PAWP were also significantly lower in the intervention group (*p* < 0.001 and *p* = 0.042), suggesting a substantial reduction in right‐sided cardiac preload and afterload. Cardiac index was higher in the intervention group (2.6 ± 0.8 L/min/m^2^ vs. 2.2 ± 0.6 L/min/m^2^, *p* = 0.049), reflecting improved global cardiac output.

CMR imaging revealed favorable changes in RV function among patients in the intervention group. RVSV and RV stroke volume index (RVSVI) were significantly greater compared with controls (*p* = 0.048 and *p* = 0.032, respectively), while RVESV was lower (*p* = 0.038), indicating enhanced systolic function. Although RVEF was numerically higher in the intervention group (36.8% vs. 31.2%), the difference did not reach statistical significance (*p* = 0.109). Other structural parameters, such as RVEDV, RV mass, and the RV mass‐to‐volume ratio (RV mass/RVEDV), showed no significant differences between groups.

For LV function, no significant between‐group differences were observed in LVSV, LV ejection fraction (LVEF), or LV mass (*p* > 0.05), indicating that the intervention primarily affected RV‐related indices.

Regarding heart failure biomarkers, NT‐proBNP levels were significantly lower in the intervention group (198 ± 166 pg/mL vs. 406 ± 212 pg/mL, *p* = 0.004), suggesting a reduction in myocardial wall stress. Functional capacity, assessed by the 6MWD, was significantly higher in the intervention group (458 ± 71 m vs. 410 ± 66 m, *p* = 0.007), reflecting meaningful improvements in exercise tolerance and daily functioning.

No significant differences were observed in resting heart rate or hemoglobin levels between groups (*p* = 0.604 and *p* = 0.137, respectively), indicating no apparent systemic or safety‐related effects from the intervention.

### 3.3. Comparison of Cardiac Output, Stroke Volume, and Oxygen Transport Variables

At Week 6, the intervention group demonstrated superior improvements in multiple cardiopulmonary performance parameters compared to the control group (Table 2). Although resting cardiac output (both supine and upright) and peak exercise cardiac output were not significantly different between groups (*p* > 0.05), the change in cardiac output from upright rest to peak exercise was significantly greater in the intervention group (3.80 ± 1.71 L/min vs. 2.70 ± 1.81 L/min, *p* = 0.025), suggesting enhanced cardiac reserve in response to physical exertion.

Similarly, calculated stroke volume by RHC (SV_RHC_) at rest and peak did not differ significantly between groups. However, the difference in SV_RHC_ between peak exercise and resting upright state was significantly greater in the intervention group (6.8 ± 14.9 mL/beat vs. 1.3 ± 15.4 mL/beat, *p* = 0.027), further supporting a more favorable adaptation in stroke volume response during exertion.

In terms of oxygen transport capacity, the intervention group had significantly higher arterial oxygen content (18.5 ± 2.1 mL/dL vs. 17.1 ± 1.8 mL/dL, *p* = 0.015) and oxygen delivery (1650 ± 410 mL/min vs. 1388 ± 375 mL/min, *p* = 0.022) than the control group. These differences likely contributed to improved systemic oxygenation during activity. Additionally, the intervention group achieved a significantly higher percentage of predicted peak oxygen uptake (VO_2_ peak) (84.3% ± 12.1% vs. 71.2 ± 13.4%, *p* = 0.001), indicating enhanced exercise capacity and cardiopulmonary efficiency.

Furthermore, hemodynamic indicators of pulmonary vascular function also favored the intervention group. PVR was significantly lower in the intervention group compared to controls (415 ± 265 vs. 586 ± 310 dyn·s/cm^5^, *p* = 0.040), and pulmonary artery compliance was significantly higher (2.12 ± 0.46 mL/mmHg vs. 1.76 ± 0.42 mL/mmHg, *p* = 0.009), reflecting improved right ventricular afterload and pulmonary arterial elasticity.

### 3.4. Psychological Stress and Emotional Status

At baseline, there were no statistically significant differences between the intervention and control groups in measures of psychological stress or emotional symptoms, as assessed by the HAMA, the HAMD, and the SPBS. Mean HAMA scores were 17.64 ± 5.20 in the intervention group and 17.91 ± 4.87 in the control group (*p* = 0.839), while HAMD scores were 19.20 ± 6.12 and 18.78 ± 5.94, respectively (*p* = 0.796). SPBS scores were similarly comparable between groups (31.45 ± 6.84 vs. 30.98 ± 7.02, *p* = 0.808), indicating a balanced baseline in terms of psychological burden and emotional distress (Table 6).

**TABLE 6:** Comparison of psychological stress assessment between the two groups (*x* ± *s*).

Times	Group	HAMA (points)	HAMD (points)	SPBS (points)
Before intervention	Intervention group (*N* = 25)	17.64 ± 5.20	19.20 ± 6.12	31.45 ± 6.84
	Control group (*N* = 25)	17.91 ± 4.87	18.78 ± 5.94	30.98 ± 7.02
	*t*	0.204	0.26	0.245
	*p*	0.839	0.796	0.808

6 weeks after intervention	Intervention group (*N* = 25)	11.28 ± 3.94	11.36 ± 4.32	18.04 ± 4.83
	Control group (*N* = 25)	14.22 ± 4.06	15.26 ± 4.89	23.02 ± 5.44
	*t*	2.579	2.658	3.472
	*p*	0.013^∗^	0.011^∗^	0.001^∗^

^∗^ *p* < 0.05.

However, after 6 weeks of intervention, significant improvements were observed in the intervention group across all three domains. Compared with the control group, the intervention group exhibited significantly lower postintervention HAMA scores (11.28 ± 3.94 vs. 14.22 ± 4.06, *p* = 0.013), HAMD scores (11.36 ± 4.32 vs. 15.26 ± 4.89, *p* = 0.011), and SPBS scores (18.04 ± 4.83 vs. 23.02 ± 5.44, *p* = 0.001, Table 6). These results suggest that the combined intervention of MBSR and CR was effective not only in alleviating anxiety and depressive symptoms but also in reducing patients’ perceived psychological burden.

### 3.5. Safety and Tolerability

Throughout the 6‐week intervention period, no serious adverse events were reported in either group. Importantly, no patients experienced intervention‐related major complications, such as acute right heart failure, severe arrhythmias, or hemodynamic decompensation. Minor transient symptoms, including postexercise fatigue and mild musculoskeletal discomfort, were occasionally observed, predominantly following exercise sessions. These symptoms were self‐limiting, required no medical intervention, and did not interfere with protocol adherence or training progression.

Both groups demonstrated good overall treatment tolerability and safety, with high compliance rates and no withdrawals due to adverse effects. These findings support the feasibility of implementing combined MBSR and CR interventions in patients with PAH under supervised conditions.

## 4. Discussion

This randomized controlled trial investigated the effects of combining MBSR with conventional CR in patients with PAH. The intervention was designed to address both physiological impairment and psychological distress, which are frequently coexistent in PAH and may interact to exacerbate disease progression and reduce quality of life. The results demonstrated that the combined intervention led to significantly greater improvements in hemodynamic parameters, cardiopulmonary performance, exercise capacity, and psychological well‐being compared with standard rehabilitation alone. Specifically, the intervention group showed greater reductions in PVR, mPAP, and mRAP, along with a corresponding increase in cardiac index, indicating improved right ventricular load conditions. Imaging findings supported enhanced right ventricular function, while improvements in oxygen delivery and peak oxygen uptake reflected superior systemic and pulmonary adaptation to exercise. Notably, participants receiving the integrated intervention also reported significantly lower levels of anxiety, depression, and self‐perceived burden, highlighting the added value of MBSR in alleviating psychological comorbidities. Collectively, these findings suggest the potential feasibility and clinical promise of a dual‐modality, mind‐body approach, warranting further validation in larger confirmatory trials.

CR has been increasingly recognized as an effective adjunctive therapy for PAH, capable of improving not only functional capacity and quality of life but also key hemodynamic parameters. Several studies have demonstrated that structured rehabilitation programs lead to meaningful reductions in PVR and pulmonary arterial pressures, along with improvements in RV function and cardiac output [11, 28–30].

Our study extends these findings by demonstrating that a 6‐week intervention combining CR with MBSR resulted in significantly greater improvements in hemodynamics than rehabilitation alone. Specifically, the intervention group exhibited a more pronounced decrease in PVR, mPAP, sPAP, and mRAP, along with a significant increase in cardiac index, suggesting enhanced RV–pulmonary artery coupling and improved RV performance. These findings are consistent with prior reports highlighting the potential of rehabilitation to modulate RV remodeling and afterload [28, 29, 31].

Beyond mechanical adaptation, improvements may also involve autonomic and vascular mechanisms. MBSR has been shown to attenuate sympathetic activation and enhance parasympathetic tone, potentially improving vascular endothelial function and cardiopulmonary regulation [32]. These neurohumoral pathways may partly explain the synergistic effects observed between MBSR and CR in the present study.

Importantly, the improvement in stroke volume and stroke volume index observed via CMR imaging in our study supports the hypothesis that rehabilitation facilitates favorable RV remodeling. These results align with previous investigations reporting reduced RVESV and improved stroke indices following exercise interventions in PAH populations [6, 30]. Furthermore, our observation of increased pulmonary arterial compliance and improved cardiac output reserve during peak exercise suggests enhanced right‐sided vascular dynamics and cardiopulmonary adaptation under stress, reinforcing the physiological relevance of rehabilitation‐derived hemodynamic changes.

In addition to the mechanical effects of exercise‐based rehabilitation, the integration of MBSR may have contributed to hemodynamic improvement via indirect mechanisms. Although direct evidence on the hemodynamic effects of mindfulness in PAH is limited, it is well established that psychological stress and anxiety, common comorbidities in PAH, can negatively influence cardiovascular regulation through autonomic imbalance and heightened sympathetic activity [4, 5, 11]. By alleviating anxiety and depressive symptoms, as reflected by significant reductions in HAMA, HAMD, and SPBS scores in the intervention group, MBSR may have mitigated neurohumoral stress responses, thereby stabilizing hemodynamic fluctuations and improving vascular tone.

Previous studies have suggested that psychological distress may modulate hemodynamic parameters via neuroendocrine and inflammatory pathways [5, 31]. MBSR, through its impact on emotional regulation and stress resilience, may attenuate these maladaptive responses, offering a plausible explanation for the augmented hemodynamic benefit observed in our study. Additionally, improvements in sleep quality and overall well‐being attributed to mindfulness practices may further support systemic cardiovascular recovery, though such parameters were not directly measured in our trial.

Another important consideration is patient adherence. Enhanced self‐awareness and emotional engagement fostered by mindfulness may have improved adherence to the rehabilitation protocol, thereby amplifying its physiological impact. This interpretation is supported by earlier reports noting the importance of training adherence in optimizing CR outcomes [33].

Despite these promising results, it is important to note that the hemodynamic response to exercise in PAH may vary depending on comorbidities such as heart failure or the presence of advanced RV dysfunction [34, 35]. While our findings are robust within a clinically stable cohort, the generalizability to more severe or unstable PAH phenotypes requires further investigation.

RV function is also a pivotal determinant of prognosis in patients with PAH [36, 37]. Due to the RV’s complex geometry and sensitivity to afterload, CMR imaging has emerged as the gold standard for evaluating RV structure, function, and hemodynamics in this population [36–39]. CMR‐derived metrics such as RVSV, RVEF, RVESV, and myocardial mass are strongly correlated with disease severity, treatment response, and long‐term outcomes [37, 38, 40].

Our findings demonstrate that the combination of CR and MBSR led to significant improvements in multiple CMR parameters compared with rehabilitation alone. Specifically, patients in the intervention group showed increased RVSV and RVSVI, as well as reduced RVESV, suggesting enhanced RV systolic performance. While RVEF showed a numerical trend toward improvement, it did not reach statistical significance, possibly due to interindividual variability or the relatively short intervention duration. These results align with prior evidence indicating that exercise‐based interventions can improve RV function through reverse remodeling and enhanced RV–pulmonary arterial coupling [8, 41, 42].

Although direct investigations of MBSR’s impact on RV structure are lacking, it is plausible that reductions in psychological stress—demonstrated by our observed improvements in HAMA, HAMD, and SPBS scores—may indirectly contribute to better RV performance. Chronic psychological stress has been shown to exert deleterious effects on cardiovascular structure and autonomic regulation, potentially exacerbating RV dysfunction through heightened sympathetic activity and systemic inflammation [42, 43]. By mitigating these stress‐related responses, MBSR may foster a more favorable milieu for cardiopulmonary recovery, although further mechanistic studies are warranted.

CMR’s role in PAH extends beyond simple volumetric assessments. It allows for detailed evaluation of myocardial strain, ventricular–vascular interaction, and pulmonary arterial flow dynamics—including novel 4D‐Flow MRI approaches that reflect diastolic dysfunction and intraventricular blood flow abnormalities [44–46]. Although our study did not incorporate advanced strain imaging or flow mapping, the favorable changes in volumetric indices strongly suggest beneficial effects on RV workload and contractile reserve.

Existing studies on post‐thromboembolic pulmonary hypertension and other cardiopulmonary conditions have reported CMR‐detectable improvements in cardiac morphology following rehabilitation [47], though such data remain limited in PAH populations. Our study is among the first to demonstrate that integrating MBSR with structured CR can induce measurable improvements in RV volumetric function, as assessed by CMR.

In summary, this pilot randomized controlled trial provides preliminary evidence that integrating MBSR with CR may enhance cardiopulmonary function and psychological resilience in PAH patients. While these results are encouraging, they should primarily be viewed as hypothesis generating. Future large‐scale studies are essential to confirm these findings and to determine the optimal implementation strategies in routine care.

Future investigations should build upon these findings by incorporating longitudinal CMR follow‐ups, myocardial strain analyses, and machine learning–based image quantification to delineate subtle myocardial and vascular adaptations. Moreover, the potential synergistic effects of psychological interventions on myocardial energetics and structural plasticity deserve focused exploration, particularly in the context of chronic right heart overload and neurohumoral dysregulation in PAH.

Several limitations should be acknowledged. First, the relatively small sample size and short follow‐up period limit the generalizability of our findings. Second, although randomization minimized selection bias, residual confounding cannot be entirely excluded. Third, the study was not powered to detect long‐term clinical outcomes. Therefore, our findings should be interpreted as preliminary evidence of feasibility and efficacy, serving as a foundation for larger multicenter randomized trials.

In conclusion, this study demonstrates that a 6‐week CR and mindfulness intervention is both effective and safe in improving cardiopulmonary function, oxygen transport, and psychological well‐being in patients with PAH. These findings reinforce the critical role of comprehensive, multidisciplinary approaches in PAH management and provide a rationale for broader clinical adoption and policy support for structured rehabilitation programs in this high‐risk population.

## Author Contributions

Jingjuan Qian conceived the study, designed the protocol, performed data acquisition, and drafted the manuscript. Chunjie Song contributed to patient enrollment and clinical data collection and assisted in data interpretation and manuscript revision. Meng Yuan performed data curation and statistical analyses and contributed to the preparation of tables and figures. Yanfeng Bai assisted with literature review, quality control of the dataset, and interpretation of results. Dan Li contributed to methodology refinement, statistical verification, and critical revision of the manuscript for important intellectual content. Yani Chen provided overall supervision, secured clinical/administrative support, guided the study design and interpretation, and finalized the manuscript. Yani Chen served as the corresponding author and takes responsibility for the integrity of the work as a whole.

## Funding

This study was supported by Suqian Sci&Tech Program (Grant no. KY202204).

## Conflicts of Interest

The authors declare no conflicts of interest.

## Data Availability

The data that support the findings of this study are available from the corresponding author upon reasonable request.
